# Recurrent laryngeal nerve monitoring during totally robot-assisted Ivor Lewis esophagectomy

**DOI:** 10.1007/s00423-020-01990-0

**Published:** 2020-09-24

**Authors:** J. I. Staubitz, P. C. van der Sluis, F. Berlth, F. Watzka, F. Dette, A. Läßig, H. Lang, T. J. Musholt, P. P. Grimminger

**Affiliations:** 1 Department of General, Visceral and Transplantation Surgery, University Medical Center Mainz, Johannes Gutenberg University Mainz, Langenbeckstraße 1, 55131 Mainz, Germany; 2 Department of Anesthesiology, University Medical Center, Johannes Gutenberg University Mainz, Langenbeckstraße 1, 55131 Mainz, Germany; 3 Communication Disorders Division, Department of Otorhinolaryngology, University Medical Center Mainz, Johannes Gutenberg University Mainz, 55131 Mainz, Germany; 4 Section of Endocrine Surgery, Department of General, Visceral and Transplantation Surgery, University Medical Center Mainz, Johannes Gutenberg University Mainz, Langenbeckstraße 1, 55131 Mainz, Germany

**Keywords:** Esophageal cancer, Larynx, Robot-assisted surgery, Intraoperative nerve monitoring

## Abstract

**Purpose:**

The robot-assisted approach for Ivor Lewis esophagectomy offers an enlarged, three-dimensional overview of the intraoperative situs. The vagal nerve (VN) can easily be detected, preserved, and intentionally resected below the separation point of the recurrent laryngeal nerve (RLN). However, postoperative vocal cord paresis can result from vagal or RLN injury during radical lymph node dissection, presenting a challenge to the operating surgeon.

**Methods:**

From May to August 2019, 10 cases of robot-assisted minimally invasive esophagectomy (RAMIE) with extended 2-field lymphadenectomy, performed at the University Medical Center Mainz, were included in a prospective cohort study. Bilateral intermittent intraoperative nerve monitoring (IONM) of the RLN and VN was performed, including pre- and postoperative laryngoscopy assessment.

**Results:**

Reliable mean signals of the right VN (2.57 mV/4.50 ms) and the RLN (left 1.24 mV/3.71 ms, right 0.85 mV/3.56 ms) were obtained. IONM facilitated the identification of the exact height of separation of the right RLN from the VN. There were no cases of permanent postoperative vocal paresis. Median lymph node count from the paratracheal stations was 5 lymph nodes.

**Conclusion:**

IONM was feasible during RAMIE. The intraoperative identification of the RLN location contributed to the accuracy of lymph node dissection of the paratracheal lymph node stations. RLN damage and subsequent postoperative vocal cord paresis can potentially be prevented by IONM.

## Introduction

The use of intraoperative nerve monitoring (IONM) was shown to contribute to the identification of the recurrent laryngeal nerve (RLN) in different studies analyzing thyroidectomy, esophagectomy, and mediastinal lymph node dissection [1–3]. The reliable identification of the RLN is essential for an intentional preservation of the nerve during surgery. The DaVinci Xi robotic system offers an enlarged three-dimensional view of the intraoperative situs during robot-assisted esophagectomy for distal esophageal cancer, facilitating the identification of the vagal nerve (VN) without the support of IONM. Yet, the exact separation point of the RLN from the VN can be difficult to distinguish from the surrounding tissues, especially in cancer patients following neoadjuvant treatment. Vagal injury above this very point and damage of the RLN can lead to the development of vocal cord paresis (VCP). The risk of intraoperative damage is particularly high during lymphadenectomy along the RLN lymph node chain. Depending on the mechanism of injury, VCP can be transient or permanent. VCP is associated with a postoperatively elevated risk of aspiration pneumonia and voice impairment [4, 5]. Furthermore, bilateral VCP can lead to a mechanically based respiratory insufficiency, which potentially requires tracheotomy [4, 5].

In open thoracic esophagectomy with extended 2-field lymphadenectomy, the use of RLN monitoring was described as a useful method, which leads to a reduction of cases with postoperative vocal cord paresis [3, 6]. In minimally invasive (thoracoscopic) esophagectomy, the use of IONM for RLN detection before visual contact (“mapping”) was proven to be associated with a reduction of the postoperative vocal cord paresis rate, when compared with esophagectomy without IONM support [7].

Nowadays, the interest in the use of robot-assisted minimally invasive esophagectomy (RAMIE) for esophageal cancer is increasing [8–10]. As an advantage over the aforementioned techniques, RAMIE allows for the dissection with 7 degrees of freedom, which is beneficial for a precise dissection of the esophagus and associated lymph node stations in the upper mediastinum [11, 12]. Moreover, a more radical resection of upper mediastinal lymph nodes is facilitated by RAMIE, when compared with minimally invasive esophagectomy [13–16].

To our knowledge, the use of IONM in robot-assisted esophagectomy was not described before. The aim of this study was to document the feasibility of the IONM technique during a standardized totally robot-assisted Ivor Lewis esophageal resection with an extended 2-field lymph node dissection.

## Material and methods

### Patients

Patients with lower esophageal cancer and a preoperatively intact vocal cord function, treated at the University Medical Center Mainz, were eligible for this prospective cohort study. Underlying histological entities were squamous cell carcinoma and adenocarcinoma with different localizations from the anterior dental row (Table 1). Patient- and treatment-related characteristics (tumor entity, TNM classification, and preoperative treatment) were prospectively collected in an institutional database. Initial staging included endoscopy combined with endoscopic ultrasonography and tumor biopsy as well as a thoracoabdominal computed tomography. Prior to treatment, all patients were discussed in an upper gastrointestinal multidisciplinary tumor board to determine optimal treatment. Standard neoadjuvant treatment for patients with esophageal adenocarcinoma was perioperative chemotherapy with FLOT (4 preoperative and 4 postoperative 2-week cycles of docetaxel 50 mg/m^2^, oxaliplatin 85 mg/m^2^, leucovorin 200 mg/m^2^, and fluorouracil 2600 mg/m^2^), or CROSS chemoradiotherapy with carboplatin (area under the curve of 2 mg per milliliter/min) and paclitaxel (50 mg/m^2^ of body-surface area) for 5 weeks and concurrent radiotherapy (41.4 Gray in 23 fractions, 5 days per week) [17, 18] (Table 1). Postoperative follow up was carried out at the department’s outpatient clinic. The study protocol conforms to the ethical guidelines of the 1975 Declaration of Helsinki (6th revision, 2008) as reflected in a priori approval by the institution’s human research committee. Informed consent was obtained from all participants.

**Table 1:** Patient characteristics

Patient	Histology	TNM Classification [41]	Preoperative treatment	Localization (cm)^a^
1	ESCC	pT3, pN2 (3/53), L0, V1, Pn0	none	30-x, stenosis
2	EAC	ypT1b, ypN3 (8/28), L0, V0, Pn0	CROSS^b^	38–41
3	EAC	ypT2, ypN0 (0/32), L0, V0, Pn0	DANTE^c^	33–37
4	EAC	ypT3, ypN1 (1/81), L0, V0, Pn0	FLOT^d^	31–42
5	ESCC	pT3, pN1 (1/22), L1, V0, Pn1	none	30–32
6	EAC	pT1a, pN0 (0/50), L0, V0, Pn0	FLOT^d^	35–42
7	EAC	ypT3, ypN3 (8/35), L0, V0, Pn0	CROSS^b^	36–42
8	ESCC	ypTis, pN0 (0/33), L0, V0, Pn0	CROSS^b^	26–32
9	ESCC	ypT0, pN0 (0/38), L0, V0, Pn0	CROSS^b^	28–30
10	ESCC	ypT3, pN0(0/30), L0, V0, Pn0	CROSS^b^	28–30

*EAC* esophageal adenocarcinoma, *ESCC* esophageal squamous cell carcinoma

^a^From anterior dental row in preoperative endoscopy

^b^CROSS (41.4Gy plus carboplatin/paclitaxel) [18]

^c^FLOT (5-fluorouracil/calciumfolinate/oxaliplatin/docetaxel) + atezolizumab, DANTE study

^d^FLOT (5-fluorouracil/calciumfolinate/oxaliplatin/docetaxel) [17]

### Technique of IONM

Intraoperative nerve monitoring of the recurrent laryngeal nerve and the vagal nerve is a standard procedure in thyroid surgery at the University Medical Center Mainz. It was newly introduced also for oncological esophagus surgery (especially RAMIE), in order to facilitate the detection of the recurrent laryngeal nerve during lymph node dissection in the upper mediastinum. Whereas for thyroid surgery continuous nerve monitoring is performed [4, 19] - allowing for an immediate feedback of an impaired nerve function due to an operation maneuver - for the present study in esophagus surgery, intraoperative nerve monitoring (IONM) was used to locate the recurrent laryngeal nerve by intermittent nerve stimulation. Nerve stimulation was performed with 7 Hz, 200 μs, 2 mA.

### Perioperative management

One day preoperatively, at the Department of Otorhinolaryngology of the University Medical Center Mainz, patients underwent a standardized videolaryngeoscopic evaluation of the vocal cord function, including photo documentation. In preparation for surgery, gastroscopy with pylorus dilatation was conducted to prevent postoperative delayed gastric emptying [20]. All patients received an epidural catheter and were intubated with a double-lumen tube. Antibiotic prophylaxis (ampicillin 2000 mg and sulbactam 1000 mg) was administered 30 min prior to incision. Postoperatively, extubation took place in the operation theater. All patients were admitted to the intensive care unit (minimum observation time: one night postoperatively). A postoperative laryngoscopy control was performed before discharge from hospital.

### Surgery

As previously described by our group, totally robot-assisted Ivor Lewis esophageal resection with extended 2-field lymphadenectomy using Da Vinci Xi (Intuitive Surgical Inc., Sunnyvale, CA, USA) was performed as a modification of the original RAMIE technique [8, 21]. This procedure was shown to be technically feasible and safe [8]. All operations were carried out by one surgeon, ensuring a high comparability between the single interventions.

### RAMIE—abdominal phase

The patient was placed in supine position. The lesser omentum was transected up to the left crus of the diaphragm. Then, the greater gastric curvature was dissected using a harmonic ace. An abdominal lymphadenectomy was performed including lymph nodes at the celiac trunk, along the left gastric and splenic artery and including the lesser omentum. The left gastric artery and vein were transected following ligation with Hem-o-lok (Teleflex Medical, Weck Driv, NC, USA).

### RAMIE—thoracic phase

**Fig. 1:**
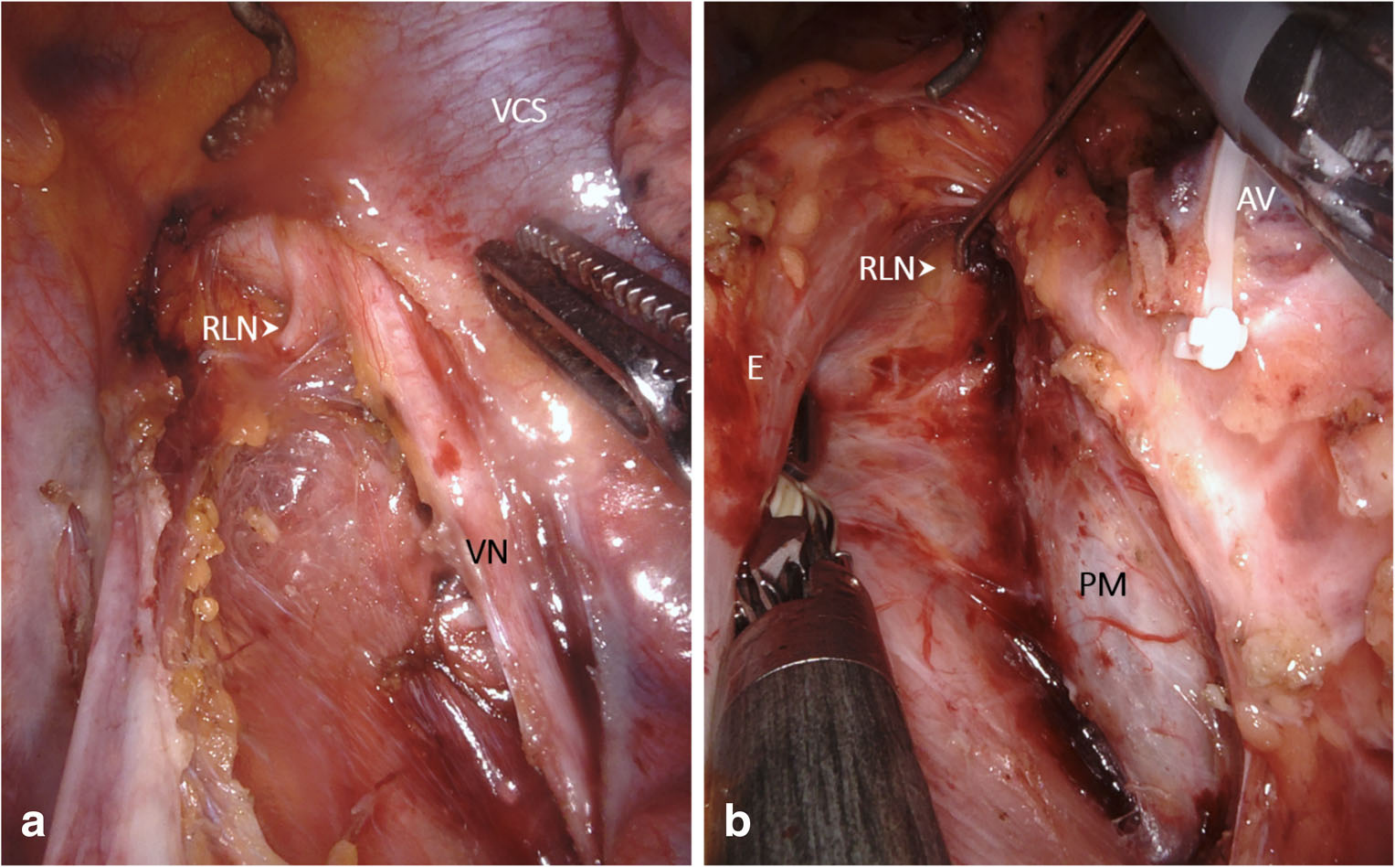
Intraoperative situs during robot-assisted Ivor Lewis esophagectomy (RAMIE). **a** Intraoperative situs with right recurrent laryngeal nerve and vagal nerve. **b** Situs with left recurrent laryngeal nerve during intermittent intraoperative nerve monitoring. Abbreviations: AV azygos vein, E esophagus, VN vagal nerve, RLN recurrent laryngeal nerve, VCS superior vena cava, PM trachea, pars membranacea

The patient was positioned in the left lateral decubitus position, tilted 45° towards the prone position. The robotic system was located at the dorsocranial side of the patient. Four ports were placed for the robot-assisted system and one thoracoscopic port for the assisting surgeon. After selective desufflation of the right lung, the pulmonary ligament was divided. Then, the parietal pleura was dissected at the anterior side of the esophagus from the diaphragm up to the azygos arch. The azygos vein was transsected following ligation with Hem-o-lok (Teleflex Medical, Weck Driv, NC, USA). Dissection of the parietal pleura was continued above the azygos arch for a dissection of the right paratracheal lymph nodes. Intraoperative nerve monitoring of the right VN and RLN was performed (Fig. 1a). At the posterior side of the esophagus, the parietal pleura was dissected from cranially to caudally along the azygos vein, including the thoracic duct. The thoracic duct was clipped with a 10-mm endoscopic clipping device (EndoclipTM II; Covidien, Mansfield, Massachusetts, USA). Lymph node dissection was continued in the left paratracheal region, complemented by IONM “mapping” of the left RLN (Fig. 1b). The esophagus was resected en bloc with the surrounding mediastinal and paratracheal lymph nodes. The resected specimen contained right-sided paratracheal (lymph node station 2R), left-sided paratracheal (lymph node station 2L) tracheobronchial (lymph node station 4), aortopulmonary window (station 5), and carinal (station 7) and peri-esophageal (station 8) lymph nodes [22, 23].

### RAMIE—application of IONM

**Fig. 2:**
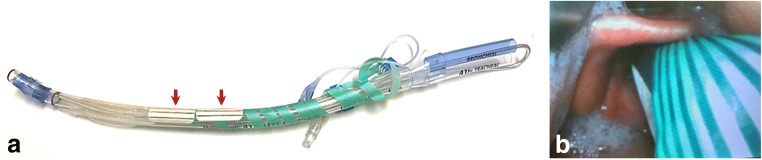
Preparation of double-lumen tube with non-invasive surface electrodes. The endotracheal double-lumen tube is prepared with non-invasive surface electrodes (red arrows, **a**), which during intubation using video laryngoscopy (C-MAC®, Karl Storz SE & Co. KG, Tuttlingen, Germany) are positioned at the vocal cord level (**b**)

**Fig. 3:**
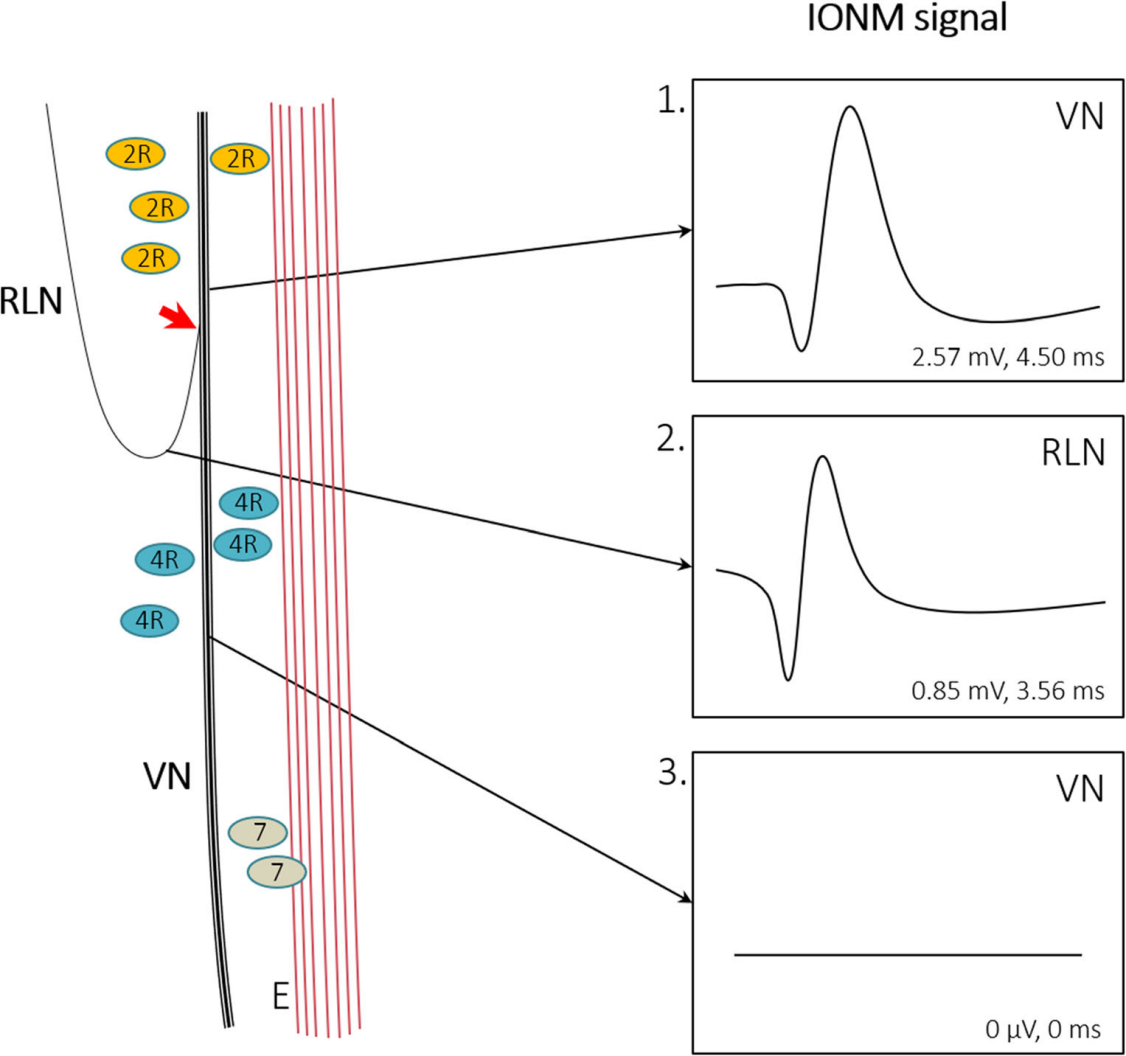
Scheme of intraoperative nerve monitoring (IONM) signals from the right vagal nerve (VN) and recurrent laryngeal nerve (RLN). IONM superior to the separation of the RLN from the VN (red arrow) leads to typical VN signals (1). The signal resulting from RLN stimulation (2) showed insignificantly lower amplitudes than the signal resulting from VN stimulation (1). VN stimulation after the separation from the RLN does not lead to measurable signals in IONM (3). Mean signal characteristics resulting from the current investigation are presented. Abbreviations: E esophagus, IONM intraoperative nerve monitoring, VN vagal nerve, RLN recurrent laryngeal nerve, 2R lymph node station 2R according to [24], 4R lymph node station 4R according to [24], 7 lymph node station 7 according to [24]

IONM signal registration was facilitated by non-invasive endotracheal tube surface electrodes (Inomed GmbH, Emmendingen, Germany), attached to the double-lumen tube (Mallinckrodt, Covidien, Dublin, Ireland: Fig. 2a). During intubation, the electrodes were positioned at the vocal cord level (Fig. 2b). A bipolar stimulation probe (7 Hz, 200 μs, 2 mA, Inomed GmbH, Emmendingen, Germany) was introduced into the thoracic situs via an assistant port located in the fourth intercostal space, posterior axillary line. The stimulation probe was robotically positioned for intermittent nerve monitoring of the VN and for mapping of the RLN. IONM preceded the mobilization of the cranial portion of the esophagus and lymphadenectomy in the upper and lower paratracheal lymph node stations 2R, 2L, and 4 (Fig. 3). If required for a modification of the resection strategy, IONM mapping was repeated. In cases of particularly distal esophageal resection, singularly the left and right RLN were assessed. In these cases an assessment of the VN was not performed, since the additional preparation of the VN cranially to the operation site might have provoked unnecessary damage. Contemporaneous assessment of amplitude and latency was performed to characterize RLN and VN signals.

## Results

In 10 patients with esophageal cancer, a totally robot-assisted 4-arm Ivor Lewis esophagectomy (RAMIE) with extended 2-field lymphadenectomy and additional IONM was performed. Mean operation time was 326 ± 37 min. Mean operation time for the thoracic phase was 233 ± 25 min. The registration of IONM signals required between 2 and 4 min of the total operation time. IONM signals before esophageal resection are presented in Table 2. For the right VN, a mean signal amplitude of 2.57 mV was calculated, with a mean latency of 4.50 ms (Table 2). An assessment of the left VN was not performed, since an additional preparation without benefit for the curative resection procedure would have been necessary. The mean values for amplitude and latency did not show significant differences between left and right RLN (mean amplitude 1.24 mV left, 0.85 mV right, mean latency 3.71 ms left, 3.56 ms right: Table 2). Tables 2 and 3 give an overview of the IONM signals obtained in the present study. The minimal differences in amplitudes and latency were caused by anatomical variation and differences in the preparation status of the tissue surrounding the analyzed nerves.

**Table 2:** Intraoperative nerve monitoring characteristics

Patients	Signal before resection							
	Vagal nerve				Recurrent laryngeal nerve			
	Left		Right		Left		Right	
	Amplitude (mV)	Latency (ms)	Amplitude (mV)	Latency (ms)	Amplitude (mV)	Latency (ms)	Amplitude (mV)	Latency (ms)
1	–	–	0.30	4.10	1.20	4.20	0.33	3.90
2	–	–	–	–	1.62	3.80	1.20	3.20
3	–	–	–	–	0.40	3.20	0.38	4.40
4	–	–	0.52	3.50	0.19	3.70	0.52	3.30
5	–	–	0.96	3.70	2.52	3.50	0.88	3.60
6	–	–	13.90	9.90	0.80	3.50	0.55	3.30
7	–	–	1.35	3.10	1.31	3.20	1.35	3.10
8	–	–	0.55	4.10	0.60	4.20	0.63	3.90
9	–	–	1.97	4.40	2.04	4.30	2.09	3.70
10	–	–	1.00	3.20	1.74	3.50	0.58	3.20
Mean ± SD	–	–	*2.57* ± 4.61	*4.50* ± 2.23	*1.24* ± 0.71	*3.71* ± 0.41	*0.85* ± 0.55	*3.56* ± 0.42

*SD* standard deviation

**Table 3:** Histopathological lymph node count

Patients	1	2	3	4	5
	Lymph node count total (N)	Lymph nodes from lymph node stations with anatomical relation to the recurrent laryngeal nerve, 2R + 2L + 4 (*N*) [24]	Percentage of lymph nodes with anatomical relation to the recurrent laryngeal nerve of total lymph node count; 2/1 (per cent)	Lymph nodes affected by tumor in 1 (*N*, per cent of 1)	Lymph nodes affected by tumor in 2 (*N*, per cent of 2)
1	53	14	26	3, 6	0, 0
2	28	4	14	8, 29	3, 75
3	32	4	13	0, 0	0, 0
4	81	20	25	1, 1	0, 0
5	22	3	14	1, 5	0, 0
6	50	12	24	0, 0	0, 0
7	35	3	9	8, 23	2, 67
8	33	6	18	0, 0	0, 0
9	38	6	16	0, 0	0, 0
10	30	3	10	0, 0	0, 0
Mean ± SD	40 (±16)	11 (±6)	27	2(±3)	1 (±1)
Median	34	5	15	1	0

*SD* standard deviation

IONM facilitated the secure resection of upper and lower paratracheal lymph nodes in regions 2R, 2L, and 4 according to the American Thoracic Society [24]. The mean number of resected lymph nodes on final histology was 40 (median lymph node count 34). On average, 27% of these originated from the lymph node stations 2R, 2L, and 4 (median lymph node count 5). The postoperative laryngoscopy assessment showed normal vocal cord function in all cases.

## Discussion

Nowadays, the rate of postoperative vocal cord paresis is considerable in esophagus surgery: from 1% up to 45.3% of patients undergoing esophagectomy (for squamous cell carcinoma, adenocarcinoma, and other cancer types) were reported to suffer from this complication [6, 25–28]. The VCP rate can be reduced by the choice of approach (open versus minimally invasive) and is dependent on the location of anastomosis [28]. For thyroid surgery, in which IONM represents a quality standard [4, 29], a rate of permanent VCP of roughly 1% is observed. Yet, thyroid redo-surgery leads to higher rates of transient VCP in up to 12.5% and permanent lesions in 3.8% [30]. In esophageal surgery, the use of intraoperative nerve monitoring was shown to be associated with a reduction in the postoperative VCP rate, e.g., from 9.8 to 0% (*p* = 0.029) in open surgery and 32.1 to 9.7% (*p* = 0.03) in minimally invasive esophagectomy [6, 7]. In 2012, Zhong et al. reported an algorithm for IONM in open thoracic esophagectomy. "Step 1" refers to testing the complete neural arch (VN + RLN) by stimulating the VN with signal retrieval at the vocal cord level. "Step 2" comprises the direct stimulation of the RLN. "Step 3" is a control of the RLN signal after resection, and "step 4" extends the post-resection control to the complete neural arch by another stimulation of the VN. This algorithm is similar to the systematic procedure recommended for IONM in thyroid and parathyroid surgery, which was published by the International Neural Monitoring Study Group in 2011 [31]. Yet, a pre- and postoperative laryngoscopy control was additionally recommended by Randolph et al. [31, 32]. In the present study on IONM during totally robot-assisted Ivor Lewis esophagectomy, we have performed pre- and postoperative laryngoscopy to assess the vocal cord function. However, we did not strictly adhere to the algorithm proposed by Zhong et al. in 2014. For this study, a pre-resection stimulation of the right VN including the mapping of the right RLN preceded the resection. Yet, if required by a modification of the resection strategy, we repeated IONM, to achieve an optimum localization of the RLN during the complete process of extended 2-field lymphadenectomy (Figs. 1 and 3). Even though the robot-assisted approach offers a precise overview of the intraoperative situs, in numerous cases, the left RLN is particularly difficult to distinguish from the surrounding tissues by the naked eye, underlining the utility of IONM mapping (Fig. 1b).

In the literature, different parameters were reported to influence the postoperative VCP rate resulting from esophagectomy. The left RLN was demonstrated to be the predominant localization of RLN injury [25]. It is plausible that the underlying anatomical location of the left RLN circumventing the aortic arch is responsible for this observation. Moreover, longer operation time and an advanced patient age were identified as significant independent predictors for VCP in esophagus surgery in multivariate analysis [25]. Furthermore, the use of continuous nerve monitoring in open esophageal resection with mediastinal lymphadenectomy was described [6]. The advantage of continuous nerve monitoring lies in the early discovery of a dissociation of amplitude and latency [4, 32, 33]. The dissociation illustrates an impeding loss of signal (LOS), potentially associated with postoperative VCP. Following an observed LOS, in thyroid surgery, an intended total thyroidectomy is recommended to be terminated after hemithyroidectomy, in order to avoid the potential risk of a bilateral VCP. A two-staged operation is the result, including rest-thyroidectomy after the certification of an intact vocal cord function by laryngoscopy in the meantime. In esophageal resection, the information about an intraoperative LOS cannot lead to a two-staged procedure. Yet, lymphadenectomy in the upper mediastinum can be modified. A reduction to unilateral, paratracheal lymphadenectomy can be considered in cases of LOS. Moreover, the postoperative management can be adapted to an observed LOS, i.e., a meticulous breathing therapy to prevent pneumonia can be exerted. VCP was demonstrated to significantly influence the risk of hospital readmission, dysphagia, hospitalization for lower respiratory tract infection, and gastrostomy/tracheostomy tube placement [5]. Since conventional continuous IONM with direct vagal stimulation would require an additional port during robot-assisted or minimally invasive esophagectomy, the non-invasive nerve monitoring making use of the laryngeal adductor reflex, as described by Sinclair et al. [34], is of particular interest.

The surgical radicality concerning the extent of lymphadenectomy differs between western countries and Asia. Whereas in the western world a comprehensive paratracheal lymphadenectomy is not yet commonly performed, in Asia, more radical approaches are applied [6]. The incidence of esophageal squamous carcinoma (ESCC)—the primary diagnosis in Asian countries—is relatively decreasing, whereas over the last decades, an increase in the incidence of esophageal adenocarcinoma (EAC) was observed [35, 36]. This increase primarily refers to western populations [35, 37]. In comparison to ESCC, EAC is diagnosed at higher tumor stages and is associated with earlier regional lymph node dissemination, a higher percentage of invaded lymph nodes and higher diffuse recurrence rates [38–40]. As a consequence, a comprehensive lymphadenectomy will gain more and more importance in the near future, also in western countries. In Asian studies, where RAMIE was compared with MIE, a higher mean lymph node yield along the recurrent laryngeal nerve was demonstrated in favor of RAMIE [13–15]. We also experienced the more radical resection of upper mediastinal lymph nodes during RAMIE in a cohort from the University Medical Center Mainz comparing robot-assisted minimally invasive esophagectomy with conventional minimally invasive esophagectomy (MIE) in a propensity-matched analysis [16].

A shift from MIE towards RAMIE can therefore be expected. The further use of IONM, e.g., as reported by Zhong et al. in 2012 in open thoracic surgery, can contribute to a reduction of resulting vocal cord paresis rates and associated postoperative morbidity. Based on the results of the present study, at the University Medical Center Mainz, IONM is performed regularly in patients undergoing RAMIE with lymphadenectomy along the recurrent laryngeal nerve lymph node chain. We experienced the application of IONM to locate the RLN as a useful addition, allowing for a safe and precise resection of the lymph nodes in the regions 2R, 2L, and 4 according to the American Thoracic Society [24]. An analysis of a potential reduction of the postoperative VCP rate is currently under preparation.

## Conclusion

The present study illustrates that intraoperative nerve monitoring is feasible during totally robot-assisted minimally invasive esophagectomy with extended 2-field lymphadenectomy. The intraoperative identification of the location of the recurrent laryngeal nerve by IONM contributed to the accuracy of lymph node dissection of the paratracheal lymph node stations. As damage of the recurrent laryngeal nerve (and therefore vocal cord paralysis) was absent in the present cohort, it can be deduced that intraoperative nerve monitoring contributes to a prevention of the recurrent laryngeal nerve during RAMIE.

## Data Availability

All data are explicitly shown in tables.
